# Clinical application of a custom-made simple subcutaneous negative pressure drainage device for the treatment of SSI after abdominal surgery: a randomized controlled trial

**DOI:** 10.1097/JS9.0000000000003504

**Published:** 2025-10-06

**Authors:** Bingyan Liu, Junjun Liu, Yanrui Ren, Jinjian Xiang, Baolai Xiao, Yimin Sun

**Affiliations:** a Department of Nursing, The First Affiliated Hospital of Yangtze University, Jingzhou, People’s Republic of China; b Department of Gastrointestinal Surgery, The First Affiliated Hospital of Yangtze University, Jingzhou, People’s Republic of China

**Keywords:** abdominal surgery, randomized controlled trial, surgical site infection, vacuum sealing drainage

## Abstract

**Background:**

The current gold standard for managing surgical site infections (SSIs) is the vacuum-assisted closure (VAC) technique, also known as vacuum sealing drainage (VSD). However, its high cost and technical complexity necessitate specialized health care personnel for proper application and monitoring, thereby limiting its widespread clinical adoption. Building on the theoretical framework and mechanistic principles of VSD systems, we developed a simplified subcutaneous negative pressure drainage device and conducted a preliminary evaluation of its clinical efficacy and potential for broader implementation.

**Objective:**

To evaluate the clinical efficacy of a novel, custom-designed simplified subcutaneous negative pressure drainage device for the treatment of SSIs following abdominal surgery.

**Methods:**

A total of 85 patients who were diagnosed with postoperative incisional infections following abdominal surgery between August 2022 and November 2024 were enrolled as study participants. These patients were randomly allocated using a computer-generated randomization sequence into either a standard care group or an intervention group. The standard care group received standard wound care, including conventional dressing changes, whereas the intervention group was treated with a custom-designed simplified subcutaneous negative pressure drainage device. The primary endpoint was wound healing status. The secondary endpoints included the wound healing time, frequency of dressing changes, cost of wound care, duration of antibiotic therapy, length of hospital stay, serum C-reactive protein (CRP) and interleukin-6 (IL-6) levels, and patient satisfaction scores.

**Results:**

Compared with the standard care group, the intervention group demonstrated superior wound healing outcomes (*P* < 0.001). Furthermore, the intervention group demonstrated significantly shorter wound healing times, fewer dressing changes, lower wound care costs, a shorter duration of antibiotic use, and shorter hospital stays than did the standard care group (all *P* < 0.001). Posttreatment CRP and IL-6 levels were significantly lower in both groups (*P* < 0.001). Patient satisfaction scores were also significantly higher in the intervention group than in the standard care group (*P* < 0.001).

**Conclusion:**

The custom-designed, simple subcutaneous negative pressure drainage device effectively improves the healing of infected wounds after abdominal surgery. It shortens the duration of antibiotic therapy and hospital stay while reducing overall treatment costs. This device demonstrates satisfactory therapeutic efficacy and holds strong potential for widespread clinical application and promotion.

**HIGHLIGHTS**

- **Innovative Device:** Development and application of a self-made, cost-effective, and simple subcutaneous negative pressure drainage device for managing surgical site infections (SSIs) after abdominal surgery.
- **Effective SSI Management:** The device significantly reduces the incidence of SSIs, promotes wound healing, and improves postoperative recovery outcomes.
- **Randomized Controlled Trial:** A robust RCT design ensures reliable and unbiased evaluation of the device’s efficacy and safety.
- **Cost-Effective Solution:** The self-made device offers a practical and affordable alternative to commercially available negative pressure wound therapy systems, particularly in resource-limited settings.
- **Clinical Implications:** Demonstrates the potential for widespread adoption in surgical practice to reduce healthcare costs, hospital stays, and patient morbidity associated with SSIs.
- **Patient-Centered Outcomes:** Highlights improved patient satisfaction and quality of life due to faster wound healing and reduced complications.
- **Safety Profile:** Confirms the device’s safety with no significant adverse events reported during the trial.

These highlights underscore the clinical relevance and potential impact of the study on postoperative care and infection control in abdominal surgery.

## Introduction

Surgical site infection (SSI) is a prevalent complication in general surgery, particularly following emergency gastrointestinal procedures such as those for perforation, appendicitis and intestinal obstruction. These operations are often associated with intraoperative contamination. Furthermore, patient-related factors, including advanced age, comorbid conditions, elevated body mass index (BMI), surgical timing, perioperative medication use, and prolonged operative duration, significantly contribute to the high incidence of postoperative wound liquefaction and infection^[1,2]^. SSIs not only delay wound healing but also adversely affect patients’ quality of life, diagnosis and overall physical and psychological well-being^[3]^. Traditional management of SSIs involves reopening the incision, evacuating subcutaneous fluid and necrotic tissue, ensuring adequate drainage to facilitate the growth of granulation tissue and subsequently performing secondary suturing^[4]^. In recent years, the advent of vacuum sealing drainage (VSD) technology has revolutionized wound care. VSD has been shown to effectively promote wound healing and reduce the incidence of wound infections^[5]^. This technique employs specialized materials, semipermeable membranes, three-way connectors, and negative pressure suction devices to create a controlled negative pressure environment. By establishing a sealed environment over the wound surface, VSD extracts fluid, blood, and bacteria, thereby minimizing the adverse effects of wound exudates and contaminants on the healing process. Additionally, it provides a moist environment conducive to the proliferation of new tissue^[6]^. VSD has gained widespread clinical application for managing large or complex traumatic wounds, postoperative wounds, and chronic ulcers^[7,8]^. Numerous studies have demonstrated that, compared with traditional dressings, VSD significantly shortens the wound healing time and reduces infection rates^[9,10]^. Despite its efficacy, VSD is associated with several limitations. The technique is relatively expensive and technically complex. The high cost of equipment and consumables, coupled with the need for skilled medical personnel for proper operation and monitoring, restricts its accessibility. Moreover, in resource-limited settings or primary health care facilities, VSD services are often unavailable.

To overcome these limitations, we developed a simplified subcutaneous negative pressure drainage system, which was introduced into clinical practice in August 2022. In contrast with conventional VSD systems that rely on costly automated pumps and proprietary materials, our innovation employs a manually operated negative pressure bulb paired with standard suction catheters. This configuration reduces material costs by approximately 95% while preserving comparable therapeutic efficacy. The design is particularly suited for resource-constrained clinical environments. This work has been reported in line with the TITAN criteria^[11]^. Preliminary clinical outcomes have been encouraging, and this report provides a comprehensive description of the device design, clinical implementation, and therapeutic outcomes associated with this cost-effective innovation.

## Materials and methods

### Material design

**Figure 1.**
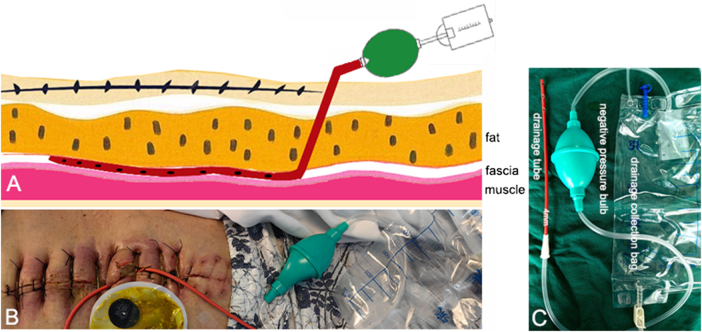
The custom-made simple subcutaneous negative pressure drainage device. (A) Schematic diagram of structure, (B) schematic diagram of operation, (C) actual object image.

The custom-designed simplified subcutaneous negative pressure drainage system comprises three primary components: a drainage tube, a manually operated negative pressure bulb, and a drainage collection bag. Specifically, the drainage tube used in this system was a disposable sputum suction catheter (manufactured by Jiangsu Jiangyang Special Rubber and Plastic Products Co., Ltd.), while the negative pressure bulb and collection bag were replaced with a single-use drainage system (supplied by Suzhou Jingle Polymer Medical Instrument Co., Ltd.; Fig. 1A and C). The side ports of the drainage tube were positioned completely beneath the skin incision. Negative pressure was maintained by manually compressing the suction bulb, thereby ensuring continuous drainage throughout the system (Fig. 1A and B).

Key innovations include (1) the use of ubiquitous sputum suction catheters and negative pressure bulbs (cost: CNY 35 vs. CNY 1200 for commercial VSD tubes; Table 1); (2) manual negative pressure bulbs eliminating electricity dependence; and (3) simplified assembly enabling bedside application without specialized training.

**Table 1:** Cost comparison between custom-made simple subcutaneous negative pressure drainage devices and commercial VSD systems

Cost component (yuan)	Custom-made device	Commercial VSD
Initial setup	35	1200
Daily maintenance	10	80
Total 7-day treatment	105	1760

### Study design

This investigation was conducted as a prospective, single-centre, randomized controlled trial. The study cohort comprised 85 patients who experienced postoperative incision liquefaction with infection following abdominal surgery between August 2022 and November 2024. This study has undergone rigorous review and has been granted approval by the hospital’s ethics committee. Furthermore, it was duly registered on chictr.org.cn. Written informed consent was obtained from all participants prior to their enrolment. The work has been reported in line with the Consolidated Standards of Reporting Trials (CONSORT) guidelines^[12]^.

### Patients

Eligible participants were adults aged 18–90 years who had undergone open gastrointestinal surgery, irrespective of sex. The inclusion criterion was the presence of characteristic local inflammatory signs (erythema, oedema, color changes, and pain) at the surgical site accompanied by visible wound liquefaction and infection^[13]^. The exclusion criteria included known hypersensitivity to drainage tube materials, current long-term corticosteroid or immunosuppressive therapy, and declining participation in the study.

### Intervention

In the standard care group, patients received conventional wound care procedures. This included routine debridement with optional wound irrigation using either hydrogen peroxide solution or 0.9% normal saline solution, followed by coverage with sterile gauze. These procedures were performed 1–2 times daily. Wound healing progression was closely monitored. For wounds exhibiting excessive exudate with inadequate drainage, the wound was left open until sufficient granulation tissue formation was observed, at which point secondary closure was performed.

The intervention group received treatment using the simplified subcutaneous negative pressure drainage system according to the following protocol:

- Initial wound debridement, with optional irrigation using hydrogen peroxide or normal saline solution, as clinically indicated.
- Placement of the drainage tube between the subcutaneous adipose tissue and fascial layer, with the distal end exiting from the lowest point of the wound.
- Fixation of the drainage tube using 2-0 silk sutures.
- Connection of the drainage tube to the negative-pressure bulb, with a hemispherical negative-pressure state maintained to establish continuous drainage.

Routine dressing changes every 48 hours, with gradual removal of the drainage tube when daily output decreased to less than 2 mL (see Supplementary Digital Content Video, available at: http://links.lww.com/JS9/F267).

Two experienced wound care nurses received standardized training on the proper application and management of negative pressure wound therapy (NPWT) systems and performed all dressing changes throughout the study period. In all patients, the device was removed prior to discharge.

### Randomization and blinding

Simple randomization was performed using SPSS. Allocation concealment was achieved through the sealed envelope method, in which randomly generated treatment assignments were placed in opaque, sealed envelopes. After obtaining the patient’s informed consent, the envelope was opened during dressing changes, and the patient was allocated to the corresponding treatment group. Both study participants and health care providers remained blinded to group allocation throughout the trial.

### Outcomes

**Figure 2.**
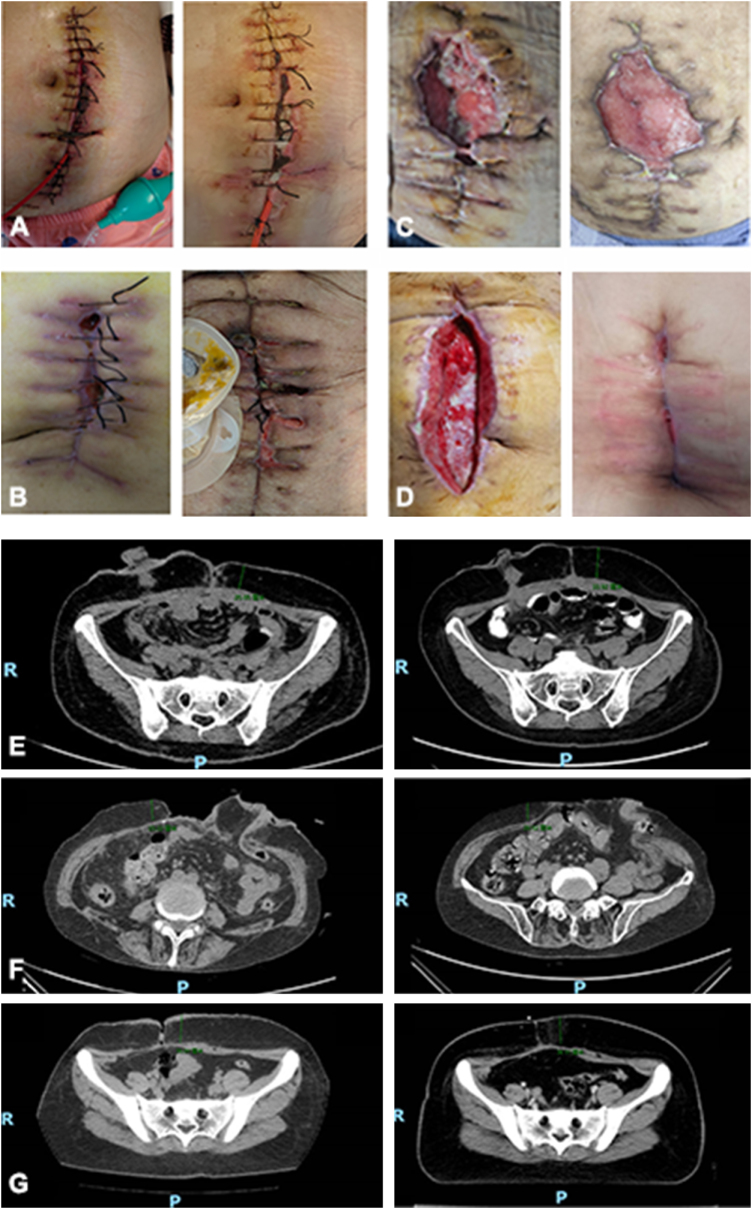
Healing conditions of SSIs. (A) Complete healing in intervention group, (B) complete healing in the standard care group, (C) re-healing after dehiscence, (D) secondary suture. Abdominal computed tomography (CT) scans: (E) complete healing, (F) re-healing after dehiscence, (G) secondary suture.

The primary outcome of this study was to compare the wound healing status of SSIs between the two groups. Healing outcomes were classified into three categories:

1) Complete healing, characterized by full epithelialization without significant tissue defects (Fig. 2A, B, and E);

2) Rehealing after dehiscence, characterized by substantial tissue defects, often associated with incisional hernia formation (Fig. 2C and F);

3) Secondary suture; characterized by wounds that required surgical reintervention due to failure of spontaneous closure (Fig. 2D and G).

The secondary outcomes included comparative analyses of the following outcomes between the two treatment groups: wound healing duration, frequency of dressing changes, total wound care costs, duration of antibiotic therapy, length of hospital stay, serial C-reactive protein (CRP) and interleukin-6 (IL-6) levels, microbial culture results from initial debridement swabs, and patient satisfaction scores.

### Follow-up

Patients underwent follow-up assessments at the wound and stoma clinic at 1, 2, and 4 weeks postdischarge. These evaluations were conducted by a wound care nurse who was not affiliated with the study and was blinded to the group assignments. At each visit, wound healing status and any complications were meticulously evaluated and documented. At 4 weeks postdischarge, a 3-point Likert scale was used to assess patient satisfaction with SSI treatment. Patients were specifically asked to indicate whether they were completely satisfied, partially satisfied, or dissatisfied with the treatment outcomes, and reasons for any dissatisfaction were carefully recorded. The outcome assessments were independently performed by a surgeon who was not part of the study team.

### Sample size

Preliminary experimental results revealed that the efficacy rates for SSI treatment were 100% in the intervention group (*n* = 20) and 75% in the standard care group (*n* = 20). For sample size estimation, we assumed SSI incidence rates of 5% for the intervention group and 30% for the standard care group. Using sample size calculation software (https://clincalc.com/stats/samplesize.aspx) with a two-tailed test at a type I error rate of 0.05 (α = 0.05), we determined that an initial sample size of 70 participants would provide 80% statistical power (β = 0.20). To account for a potential 5% dropout rate, we planned to enrol a minimum of 78 patients (39 per group) in the study.

### Statistical analysis

Normally distributed data are expressed as the mean ± standard deviation (SD) and were compared using independent samples t-tests. Nonnormally distributed variables are summarized as medians (ranges) and were analyzed using nonparametric tests: the Mann‒Whitney U test for two-group comparisons and the Kruskal‒Wallis H test for multigroup comparisons. Categorical variables are presented as frequencies and percentages (%), with intergroup differences assessed with the chi-square test (for expected cell frequencies ≥5) or Fisher’s exact test (for sparse data).

To identify independent risk factors influencing infected wound healing outcomes, a multivariate logistic regression analysis was performed, adjusting for clinically relevant covariates. Statistical significance was defined as a two-tailed *P* value <0.05. All analyses followed the CONSORT 2010 guidelines, with the per-protocol (PP) approach as the primary analysis method^[12]^. The data were visualized using GraphPad Prism 9.0 (GraphPad Software, Inc.), and the statistical computations were performed with SPSS 27.0 (IBM Corp.).

## Results

**Figure 3.**
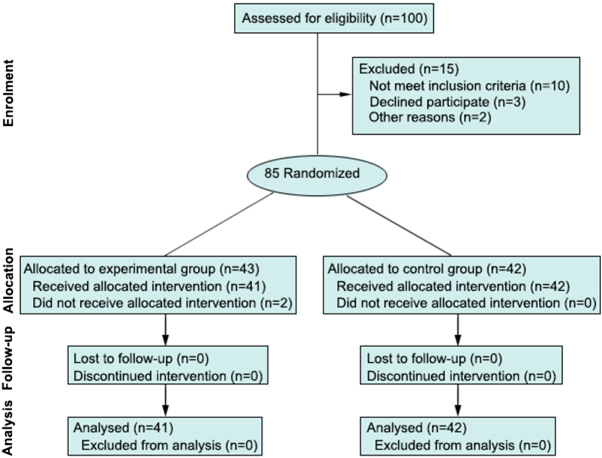
Consort flow chart.

After initial screening of 100 patients with SSIs, 15 patients who did not meet the study criteria were excluded, and 85 patients were enrolled in the study. Among the 85 patients, 2 withdrew, and no participants were lost to follow-up, resulting in 83 patients being included in the analysis (Fig. 3). Among the 83 patients, 50 were female and 33 were male; the average age was 62.4 (SD: 10.6) (range, 26–89) years. Sixteen patients (19.3%) had a history of smoking, and 17 patients (20.5%) had internal medical comorbidities (15 with diabetes, 2 with COPD). The average BMI was 23.5 kg/m^2^(SD: 2.7; range, 18–28.5), the average serum albumin level was 31.2 g/L (SD: 2.9; range, 22.8–35.7), the average incision length was 17.6 cm (SD: 4.7; range, 10–25), the average subcutaneous fat thickness was 2.9 cm (SD: 0.7; range, 1.2–4.9), and the average operation time was 226.2 minutes (SD: 92.9; range, 91–445). The American Society of Anesthesiologists (ASA) classification of the patients was Grade I in 22 cases (26.5%), Grade II in 50 cases (60.2%), Grade III in 9 cases (10.8%), and Grade IV in 2 cases (2.4%). Eight patients (9.6%) underwent gastric surgery, 30 patients (36.1%) underwent small intestine surgery, 43 patients (51.8%) underwent colorectal surgery, and 2 patients (2.4%) underwent appendectomy. As shown in Table 2, there were no significant differences between the two groups in terms of demographics, BMI, hypoproteinaemia, incision length, subcutaneous fat thickness, operation time, surgical site, or ASA classification.

**Table 2:** Baseline characteristics of patients

Index	Intervention group	Standard care group	*P*
Gender			0.754
Male	17 (41.5%)	16 (38.1%)	
Female	24 (58.5%)	26 (61.9%)	
Age (years)	62.5 ± 10.6	62.3 ± 10.7	0.915
BMI(kg/m^2^)	23.2 ± 2.8	23.8 ± 2.6	0.339
Hypoproteinemia (g/L)	31.1 ± 2.8	31.3 ± 3.0	0.785
Incision length (cm)	17.5 ± 5.1	17.6 ± 4.4	0.937
Fat thickness (cm)	2.9 ± 0.7	2.9 ± 0.8	0.930
Operation time (min)	232.0 ± 99.1	222.5 ± 87.3	0.579
Surgical sites			0.986
Stomach	4 (9.8%)	4 (9.5%)	
Small intestine	14 (34.1%)	16 (38.1%)	
Colorectal	22 (53.7%)	21 (50.0%)	
Appendix	1 (2.4%)	1 (2.4%)	
Diabetes			0.736
Yes	8 (19.5%)	7 (16.7%)	
No	33 (80.5%)	35 (83.3%)	
COPD			0.986
Yes	1 (2.4%)	1 (2.4%)	
No	40 (97.6%)	41 (97.6%)	
Smoking			0.957
Yes	8 (19.5%)	8 (19.0%)	
No	33 (80.5%)	34 (81.0%)	
ASA score			0.948
1	10 (24.4%)	12 (28.6%)	
2	26 (63.4%)	24 (57.1%)	
3	4 (9.8%)	5 (11.9%)	
4	1 (2.4%)	1 (2.4%)	

ASA, American Society of Anesthesiologists; BMI, body mass index; COPD, chronic obstructive pulmonary disease.

### Primary outcome

**Figure 4.**
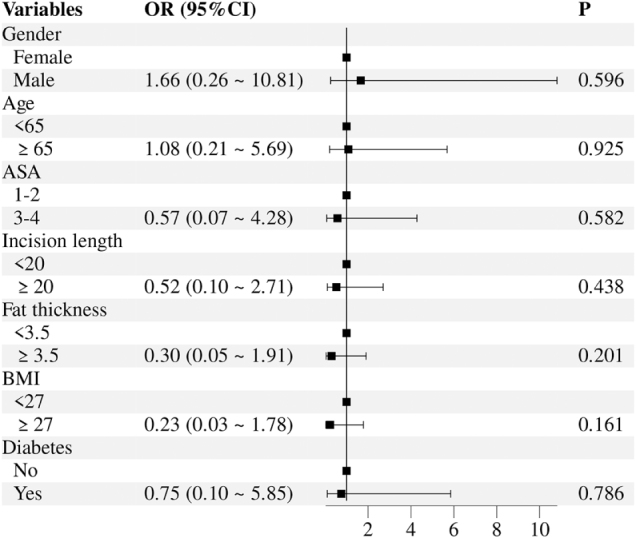
Multivariate analysis of risk factors for infected wound healing outcomes. ASA: American Society of Anaesthesiologists score; BMI: body mass index; CI: confidence interval; OR: odds ratio.

The healing status of SSIs was compared between the two groups. In the intervention group, 38 patients (92.7%) achieved complete wound healing, 3 patients (7.3%) experienced healing after wound dehiscence, and no patients required secondary suturing. In the standard care group, 11 patients (26.2%) achieved complete wound healing, 21 patients (50.0%) experienced healing after wound dehiscence, and 10 patients (23.8%) required secondary suturing. Univariate and multivariate analyses (Table 4 and Fig. 4) showed that the SSI healing rate was significantly higher in the intervention group than in the standard care group (*P* < 0.001; Table 3).

**Table 3:** Comparison of wound healing effects between intervention and standard care groups

Outcome	Intervention group (*n* = 41)	Standard care group (*n* = 42)	*P*
Wound condition			<0.001
Completely healed	38 (92.7%)	11 (26.2%)	
Healed after dehiscence	3 (7.3%)	21 (50.0%)	
Reoperation	0 (0%)	10 (23.8%)

**Table 4:** Univariate analysis of risk factors for infected wound healing outcomes

Wound healing outcomes	Yes	No	OR (95% CI)	*P*
Gender				
Male	31 (37.3%)	2 (2.4%)		
Female	42 (50.6%)	8 (9.6%)	2.95 (0.59–14.88)	0.302
Age (years)				
< 65	31 (37.3%)	6 (7.2%)		
≥ 65	42 (50.6%)	4 (4.8%)	0.49 (0.13–1.89)	0.329
ASA score				
1–2	65 (78.3%)	7 (8.4%)		
3–4	8 (9.6%)	3 (3.6%)	3.48 (0.75–16.23)	0.124
Incision length (cm)				
< 20	41 (49.4%)	5 (6.0%)		
≥ 20	32 (38.6%)	5 (6.0%)	1.28 (0.34–4.81)	0.713
Fat thickness (cm)				
< 3.5	61 (73.5%)	4 (4.8%)		
≥ 3.5	12 (14.5%)	6 (7.2%)	7.63 (1.86–31.18)	0.006
BMI (kg/m^2^)				
< 27	66 (79.5%)	5 (6.0%)		
≥ 27	7 (8.4%)	5 (6.0%)	9.43 (2.18–40.77)	0.001
Diabetes				
Yes	12 (14.5%)	3 (3.6%)		
No	61 (73.5%)	7 (8.4%)	0.46 (0.10–2.03)	0.377
COPD				
Yes	2 (2.4%)	0		
No	71 (85.5%)	10 (12.0%)	1.14 (1.05–1.24)	1.000
Smoking				
Yes	15 (18.1%)	1 (1.2%)		
No	58 (69.9%)	9 (10.8%)	2.33 (0.27–19.83)	0.687
Negative-pressure treatment				
Yes	41 (49.4%)	0		
No	32 (38.6%)	10 (12.0%)	1.31 (1.11–1.55)	0.001

ASA, American Society of Anesthesiologists; BMI, body mass index; COPD, chronic obstructive pulmonary disease.

### Secondary outcomes

Treatment outcomes differed significantly between the two groups. The intervention group had a significantly shorter mean (SD) wound healing time of 12.5 (3.8) days compared with 39.7 (19.7) days in the standard care group (*P* < 0.001). Similarly, the intervention group required fewer dressing changes—mean (SD) of 6.9 (2.0) compared with 36.8 (20.3) in the standard care group (*P* < 0.001). The total cost of dressing changes was significantly lower in the intervention group, with a median (IQR) of CNY 231.0 (198.0–280.5), compared with CNY 1353.0 (783.8–3327.6) in the standard care group (*P* < 0.001). There was a significant difference in the duration of antibiotic use between the two groups, with the intervention group having a mean (SD) duration of 9.6 (2.3) days and the standard care group having a mean (SD) duration of 16.8 (3.9) days (*P* < 0.001). There was a significant difference in the length of hospital stay between the two groups, with the intervention group having a mean (SD) of 25.7 (9.2) days and the standard care group having a mean (SD) of 52.6 (19.8) days (*P* < 0.001; Table 5).

**Table 5:** Comparison of treatment effects between the two groups $\left(\bar{\chi }\pm s\;or\;IQR\right)$

Group	Healing time (d)	Dressing time	Total dressing cost (yuan)	Antibacterial time(d)	Hospital stay(d)
Intervention group	12.5 ± 3.8	6.9 ± 2.0	231.0 (198.0–280.5)	9.6 ± 2.3	25.7 ± 9.2
Standard care group	39.7 ± 19.7	36.8 ± 20.3	1353.0 (783.8–3327.6)	16.8 ± 3.9	52.6 ± 19.8
*t*/Z	−6.076	−6.545	−7.744	−7.137	−5.493
*P*	< 0.001	< 0.001	< 0.001	< 0.001	< 0.001

**Figure 5.**
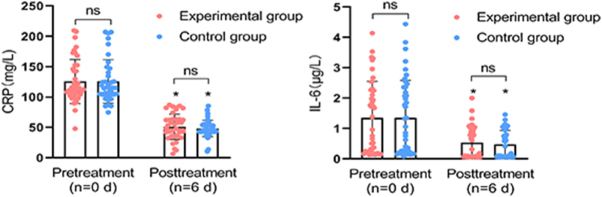
Blood CRP and IL-6 levels in the intervention group and the standard care group. *P < 0.001 vs. pretreatment. The upper limits of normal for CRP and IL-6 were defined as 6 mg/L and 0.007 μg/L, respectively.

CRP and IL-6 levels were compared between the two groups. Blood CRP and IL-6 levels were measured on the day of dressing change and on the sixth day after dressing change. The results showed no significant difference in CRP or IL-6 levels between the two groups before and after treatment. However, the CRP and IL-6 levels in both the intervention and standard care groups decreased significantly after treatment (Fig. 5).

Culturing and identification of pathogens from initial debridement swabs: Wound cultures obtained during the initial debridement were positive in 57.8% of cases, with no significant differences in pathogen distribution between the two groups (Table 6). The most commonly isolated organisms were *Klebsiella pneumoniae* subsp. *pneumoniae* (33%) and *Escherichia coli* (31%).

**Table 6:** Microbial culture of swabs from initial debridement in both groups

Microorganism	Intervention group (*n* = 41)	Standard care group (*n* = 42)	*P*
Klebsiella pneumoniae subsp. pneumoniae	9 (22.0%)	7 (16.7%)	0.919
Escherichia coli	6 (14.6%)	9 (21.4%)	
Enterococcus faecium	4 (9.8%)	3 (7.1%)	
Enterobacter cloacae	1 (2.4%)	2 (4.8%)	
Staphylococcus aureus	2 (4.9%)	3 (7.1%)	
Candida albicans	1 (2.4%)	1 (2.4%)	

Patient satisfaction was compared between the two groups. In the intervention group, 37 patients (90.2%) were completely or partially satisfied with the treatment outcome (*P* < 0.001), which was higher than the 30 patients (71.4%) in the standard care group (Table 7). Patient dissatisfaction was attributed mainly to the prolonged duration of SSI treatment and suboptimal treatment outcomes.

**Table 7:** Comparison of satisfaction between the two groups [*n*(%)]

Outcome	Intervention group (*n* = 41)	Standard care group (*n* = 42)	*P*
Satisfaction			< 0.001
Satisfied	30 (73.1%)	11 (26.2%)	
Partly satisfied	7 (17.1%)	19 (45.2%)	
Unsatisfied	4 (9.8%)	12 (28.6%)	

## Discussion

SSI is a common surgical postoperative complication, with the incidence of abdominal wound infections ranging from 8.3% to 32%^[14]^. The incidence is particularly high following colorectal surgery compared with other surgeries^[15]^. Numerous factors influence postoperative wound infections, including age, nutritional status, subcutaneous fat thickness, obesity, the degree of surgical contamination, diabetes, smoking, use of immunosuppressants, and the use of laparoscopic techniques^[14]^. Postoperative wound infections not only increase patients’ financial and psychological burden but also prolong their hospital stay^[16]^. Current clinical treatment for wound infections primarily involves traditional debridement and dressing changes, which often yield suboptimal results and may even necessitate reoperation. Studies have shown that VSD technology can significantly reduce the incidence of postoperative wound infections^[9,17]^ and is widely used in various abdominal surgeries^[18]^. The effects of VSD include rapid removal of effusion fluid, a reduction in local oedema, increased blood flow, and a decreased bacterial load in tissues^[19,20]^. Additionally, VSD improves the microcirculatory blood flow velocity in the wound, enhances reperfusion, accelerates the absorption of necrotic tissue, promotes the formation of granulation tissue, inhibits the production of inflammatory mediators and proteases, and aids in haemostasis^[21,22]^. These effects help prevent wound infections, accelerate wound healing, shorten recovery time, reduce dressing change frequency, decrease the length of hospital stay, and alleviate patients’ financial burden. Currently, clinically used VSD systems are costly and complex to operate. Based on the principles and mechanisms of such systems, we developed a simple subcutaneous negative pressure drainage device consisting primarily of a drainage tube, a hand-pressed negative pressure drainage bulb, and a drainage bag. This device is low-cost and easy to operate. Comparative studies showed that CRP and IL-6 levels were significantly lower after treatment in the intervention group, indicating that the device can reduce the production of inflammatory mediators-consistent with previous experimental findings^[23]^. The wound healing rate in the intervention group was significantly better than that in the standard care group. Moreover, the intervention group had significantly shorter wound healing times, fewer dressing changes, lower total dressing costs, shorter antibiotic use durations, and shorter hospital stays than did the standard care group. In addition, the evaluation of patient-reported outcomes revealed that satisfaction was significantly greater in the intervention group than in the standard care group. This finding is consistent with previous SSI research^[24]^. Therefore, the efficacy of our custom-made simple subcutaneous negative pressure drainage device in treating postoperative wound infections is consistent with the aforementioned research results on VSD technology. Particularly in resource-limited clinical settings where advanced NPWT systems, such as those relying on VSD, are unavailable, our device offers a clinically practical option. While the present study focused solely on the therapeutic efficacy of simplified negative pressure drainage for infected abdominal incisions, future research should evaluate its potential application in preventing SSIs among obese individuals (BMI ≥ 30). In these patients, thicker subcutaneous tissue may predispose them to postoperative abdominal incision infections. Similarly, this device could also be considered for treating incision infections following laparoscopic procedures, despite their generally lower SSI incidence rates.

For patients with postoperative wound infections treated using the custom-made simple subcutaneous negative pressure drainage device, the following precautions should be noted: First, conventional dressing changes should be performed on the infected wound before placing the negative pressure drainage device. The wound should be explored, and any effusion (pus) and necrotic tissue should be removed. Second, incision sutures should not be removed unless necessary. Third, the side-hole drainage tubes should be buried between the subcutaneous fat and fascia, with the distal end exiting from the lowest point of the skin incision. It should be secured to the surrounding sutures with sterile silk, ensuring that all side holes are buried subcutaneously to achieve an effective negative pressure seal. For longer wounds, multiple drainage tubes can be placed. Fourth, if the sealing effect is unsatisfactory, family members can be instructed to manually compress the drainage bulb every 2 hours (excluding nighttime). Fifth, when the wound dressing becomes wet or drainage is obstructed, prompt dressing changes are needed to clean the effusion (pus) and remove necrotic tissue around the drainage tube. The drainage tube can be flushed with saline as needed. Sixth, when there is no redness or effusion at the incision site and the drainage volume is less than 2 mL, the drainage tube should be gradually withdrawn (after confirming the absence of blockage). Withdraw the tube by less than 1 cm at a time until the last side hole is reached; the tube can then be fully removed.

This study has several limitations. Owing to the widespread adoption of laparoscopic techniques at our hospital, the number of SSI cases in general surgery has been relatively low, resulting in a limited sample size for this clinical trial. This may be attributed to the well-established benefits of laparoscopic surgery, including minimal invasiveness, faster recovery, and reduced wound infection rates. Consequently, our study focused primarily on open surgical approaches, as the distinct anatomical exposures and surgical trauma patterns associated with different techniques may lead to variations in postoperative outcomes and infection risks. Future studies should further evaluate the applicability of our findings to minimally invasive procedures. For this prospective study, although our sample size met statistical requirements, broader validation through multicentre studies with larger cohorts is warranted to strengthen the generalizability of our findings. Although both participants and research team members were blinded, the visible nature of the intervention may have compromised blinding for the nurses providing wound care. While this concern is partially mitigated by the use of an objective primary outcome (wound healing status), it could introduce performance bias. Patient satisfaction was evaluated using a 3-point Likert scale (satisfied/partially satisfied/dissatisfied) due to its simplicity and clinical feasibility. However, this tool lacks formal validation. Future studies should incorporate validated measures, such as the visual analogue scale (VAS), to better meet assessment needs. This study was limited to a comparison with standard wound care rather than commercial negative-pressure systems. While this design has advantages over conventional wound management, it cannot establish relative effectiveness compared with existing technologies. Given resource constraints, this represents a pragmatic choice; however, future studies will include direct randomized comparisons with commercial VAC devices.

## Conclusion

The custom-made simple subcutaneous negative pressure drainage device described in this study is low-cost, easy to operate, and has satisfactory effects in treating SSI, making it suitable for broader clinical application and integration into clinical practice.

## Research registration unique identifying number (UIN)

ChiCTR2400095052.

## Data statement statement

The datasets generated and/or analyzed during this study are available from the corresponding author upon reasonable request.
